# Evaluating the direct superior approach compared to the traditional posterior approach for hip arthroplasty: A systematic review and meta-analysis

**DOI:** 10.1016/j.jor.2025.05.062

**Published:** 2025-05-27

**Authors:** Ralph Abdallah, Mauz Asghar, Sadek Jaber, Anthony Chalfoun, Ali Ghosn, Charbel Chaiban, Hadi Soukarieh, Ahmad Chokr, Maher Ghandour, Ümit Mert

**Affiliations:** a Faculty of Arts and Science, University of Toronto, Toronto, Canada; b University of Saskatchewan College of Medicine, Saskatchewan, Canada; c Orthopaedic Surgery Department, University Hospital of Nimes, Nimes, France; d Orthopaedic Surgery Department, Lebanese University, Beirut, Lebanon; e Department of Orthopaedic Surgery, Saint Georges University Medical Center, Beirut, Lebanon; f Department of Orthopaedic Surgery, Lebanese American University Medical Center-Rizk Hospital, Beirut, Lebanon; g Department of Orthopaedic &Spine Surgery, Hospital Caritas Dominikus, Berlin, Germany; h Department of Trauma and Orthopaedic Surgery, Helios University Hospital Wuppertal, University of Witten/Herdecke, Wuppertal, Germany

**Keywords:** Direct superior approach, Posterior approach, Hip arthroplasty, Minimally invasive hip surgery

## Abstract

**Background:**

The direct superior approach (DSA) has recently emerged as a minimally invasive substitute for the posterior approach (PA) in hip arthroplasty (HA), with the potential to provide better perioperative and functional outcomes. Nevertheless, the current evidence comparing the two approaches is limited and conflicting. Hence, we carried out this study to assess the DSA compared to PA in patients undergoing HA.

**Methods:**

A comprehensive search, encompassing PubMed, Scopus, Web of Science, and Cochrane Library, was conducted from inception until April 2025. Studies that compare DSA to PA for patients undergoing HA were included. The primary outcome was the duration of hospital stay, while the secondary outcomes were incision length, functional outcomes, discharge, surgery revision rates, and complications. Mean difference (MD) with a 95 % confidence interval (C.I.) was employed for pooling the continuous variables, while the categorical data were analysed as risk ratio (RR) with a 95 % CI.

**Results:**

Twelve studies, incorporating 147,098 patients, constituted our review. Our pooled estimate favored the DSA in decreasing the duration of hospital stay (MD = −0.95, 95 % CI [-1.32 to −0.57], p = 0.001), and shortening the incision length (MD = −5.16, 95 % CI [-6.48 to −3.85], p = 0.001) compared to the PA. Furthermore, DSA showed notably lower VAS pain scores (MD = −0.39, 95 % CI [-0.69 to −0.09], p = 0.01) and a reduced risk of discharge to rehabilitation (RR = 0.55, 95 % CI [0.40 to 0.75], p = 0.001). However, our pooled analysis did not detect significant differences between the two approaches regarding functional scores, such as HOOS subscales, HHS, OHS, WOMAC, revision rate, and complications.

**Conclusion:**

DSA was associated with a lower hospital stay, shorter incision length, and lower VAS pain scores than the PA. Additionally, no substantial differences were detected regarding functional parameters or complications.

## 1 Introduction

Hip arthroplasty (HA) is one of the most frequent orthopaedic operations, usually performed on older people.^1^^,^^2^ The incidence of hip arthroplasty is expected to rise due to the increasing prevalence of hip osteoarthritis and the aging population, two factors closely linked with the increase in the surgical demand for HA.^3^ Two main approaches for HA are present, including total hip arthroplasty (THA), typically adopted for managing degenerative diseases such as osteoarthritis, and hemiarthroplasty, usually implemented for repairing femoral neck fractures.^1^^,^^2^

The worldwide economic burden of HA is substantial, with a global hip replacement market size of 7.72 billion $ in 2023 and projected to increase to 11.59 billion $ by 2032, indicating an annual growth rate of 4.7 %.^3^ Thus, substantial enhancements in surgical techniques have been made in recent years to optimize patient outcomes, reduce complications, promote early recovery, and subsequently reduce the health-related costs of HA.

The posterior approach (PA) was traditionally considered the preferred technique for HA due to its favorable surgical exposure and simple access. Nevertheless, it was linked to a higher risk of dislocation and delayed functional recovery, as this approach violates the stabilizing structures, including the iliotibial band and the short external rotators, particularly the quadratus femoris and obturator externus muscles.^4^

To overcome these limitations of the PA, the direct anterior approach (DAA) was established as an effective substitute for PA in managing HA. It revealed substantial benefits, such as lower dislocation rates, quicker mobilization, and shorter inpatient stays.^5^, ^6^, ^7^, ^8^ However, the DAA poses major challenges as it is related to heightened risks of femorally-sided revisions and demands a steep learning curve.^2^^,^^6^

Consequently, the direct superior approach (DSA) emerged as a modification of PA that provides a similar anatomical landscape for surgeons experienced with the PA, thus resulting in a small learning curve.^9^^,^^10^ DSA is a minimally invasive technique that preserves the iliotibial band, the obturator externus tendon, and the quadratus femoris muscle, resulting in lower intraoperative trauma, shorter hospitalization, and faster postoperative recovery than the PA.^4^

Notably, the current evidence comparing the DSA and PA is conflicting, and determining the surgical approach largely depends on the surgeon's preference rather than high-quality systematic evidence. In a recent meta-analysis, Shin et al. found a greater benefit for DSA over PA, such as reduced hospital stay, lower intraoperative blood loss, and shorter incision length.^1^ Several studies have been published since this meta-analysis.^11^^,^^12^ Hence, we conducted this study to update the available evidence regarding comparing DSA and PA regarding perioperative, functional outcomes, and complications.

## 2 Methods

This study was conducted in accordance with Cochrane Handbook protocols, with all findings reported in compliance with PRISMA statement requirements.^13^^,^^14^

## 3 Criteria for eligibility

Our analysis included randomized trials and observational studies of hip arthroplasty patients receiving either direct superior (experimental) or posterior approach (control). We primarily examined length of stay, with secondary outcomes including surgical metrics (incision size, operative duration, blood loss parameters, transfusion rates) and discharge characteristics. Functional outcomes were assessed using validated instruments (Harris Hip Score and modifications, WOMAC, Oxford Hip Score, HOOS subscales, and EQ-5D). We documented complications including dislocations, fractures, neurological deficits, infections, thromboembolic events, cerebrovascular accidents, mortality, and revisions. Studies utilizing other approaches, conference abstracts, animal research, and non-English literature were excluded.

## 4 Literature search

We searched PubMed, Scopus, Web of Science, and Cochrane Library from their inception through April 2025. Our search strategy combined terms related to hip arthroplasty procedures and surgical approaches, specifically: (“Total Hip Arthroplasty” OR “Total Hip Replacement” OR “Hip Prosthesis Implantation” OR “Hip Prosthesis Implantations” OR “Hip Replacement Arthroplasty” OR “Hip Replacement Arthroplasties” OR THA OR Hemiarthroplasty OR HA) AND (“Direct superior approach” OR “direct superior” OR “Superior approach” OR DSA). After importing citations into EndNote for deduplication, two independent reviewers conducted a two-phase screening process—first assessing titles/abstracts via Rayyan platform,^15^ then examining full texts against eligibility criteria. This methodical approach maximized comprehensive literature identification while reducing selection bias through dual independent review.

## 5 Data extraction

Data extraction proceeded through dual-blind assessment by independent reviewers utilizing predetermined collection templates. Extracted data encompassed study characteristics (design methodology, national context, study period, participant numbers, selection criteria, population attributes, follow-up intervals, key outcomes) and baseline participant metrics (chronological age, gender composition, BMI values, diabetic status, smoking history, ASA classification). This rigorous extraction protocol facilitated comprehensive documentation of essential variables while maintaining methodological integrity through reviewer independence.

## 6 Quality assessment

Methodological quality assessment employed domain-specific instruments based on study design. Randomized trials underwent evaluation via Cochrane Risk of Bias 2 framework,^16^ examining randomization processes, intervention adherence, data completeness, measurement reliability, and reporting selectivity. Dual independent reviewers classified each trial as demonstrating low, moderate, or high bias risk. Observational investigations were assessed utilizing Newcastle-Ottawa Scale,^17^ which evaluates methodological integrity across three domains: selection procedures, group comparability, and outcome determination. This dual assessment approach ensured appropriate design-specific quality evaluation across the included evidence base.

## 7 Data synthesis

For statistical analysis, we calculated mean differences with 95 % CIs for continuous variables and risk ratios with CIs for dichotomous outcomes. We applied DerSimonian-Laird random-effects models with significance set at p < 0.05. Heterogeneity was evaluated using chi-square testing (significant at p < 0.1) and I^2^ statistic (substantial when exceeding 50 %). To assess result stability, we conducted leave-one-out sensitivity analysis for primary outcomes. When significant heterogeneity emerged, we employed Galbraith plots to identify contributing studies. Where sufficient data existed, we performed subgroup analysis comparing hemiarthroplasty versus total arthroplasty procedures. Given the limited studies per outcome category, we assessed publication bias using Doi plots to examine effect size-Z score relationships.

## 8 Results

### 8.1 Literature search and selection process

**Fig. 1:**
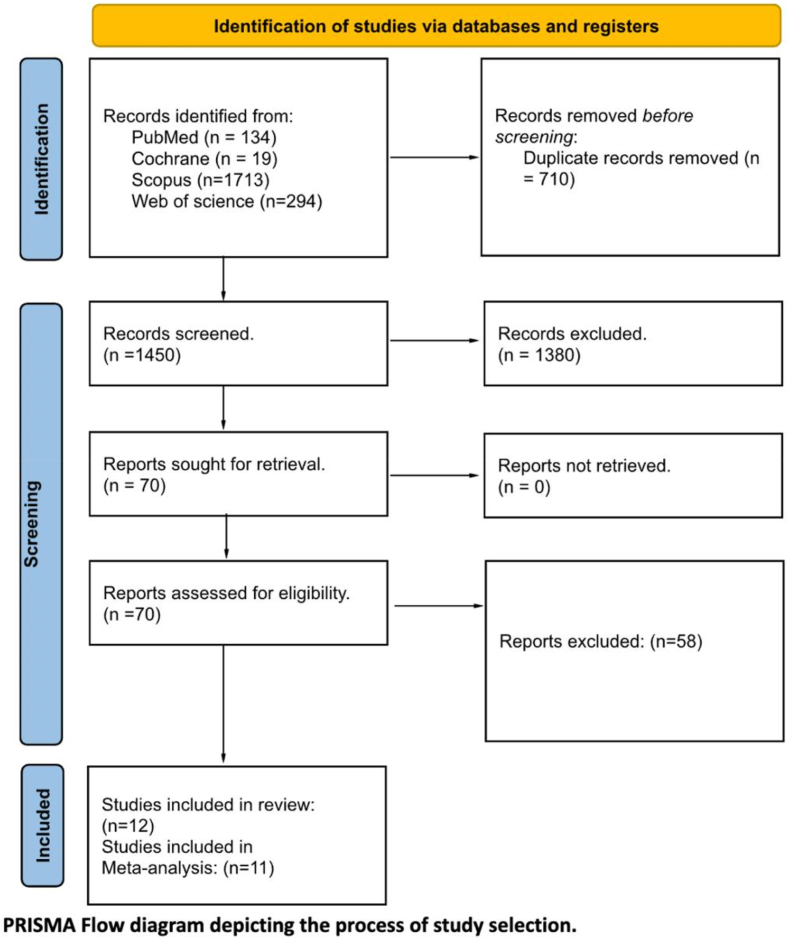
PRISMA flow diagram of the study selection process.

The literature search identified 2160 citations across four electronic databases, with 710 duplicate entries removed through Endnote software. Title and abstract screening of the remaining 1450 records eliminated 1380 irrelevant publications. Full-text assessment of 70 potentially eligible articles resulted in 58 exclusions based on predetermined criteria, yielding twelve studies for final inclusion. The systematic selection process adhered to PRISMA guidelines as depicted in Fig. 1.

### 8.2 Characteristics of the included studies

Ten observational cohorts and two RCTs, comprising 147,098 patients, were included in our study.^2^^,^^9^^,^^11^^,^^12^^,^^18^, ^19^, ^20^, ^21^, ^22^, ^23^, ^24^, ^25^ The included studies were conducted in various geographical areas: five in Europe, four in Asia, and three in North America, particularly the USA. The total hip arthroplasty was performed in ten studies, while two studies performed hemiarthroplasty. The average duration of follow-up was 17.7 months, varying from 3 to 40 months. The mean age of our study population was 66.6 [10.9] years, with a 36.1 % male gender distribution. In-depth information regarding the summary and baseline information is presented in Table 1, Table 2, respectively.

**Table 1:** Summary characteristics of the included studies.

Study ID	Study design	Country	Time frame	Sample Size			Inclusion criteria	Population type	Follow-up, months	Main results
				Total	DSA	PA				
Dooren 2023	Cohort study	Netherlands	January 2014 to December 2020	118917	1341	117576	Patients who received a primary non-metal-on-metal (MoM) THA using the DAA, PLA, or DSA.	Total hip arthroplasty	34.8	"Early nationwide results suggest that the DSA for total hip arthroplasty seems to show a tendency towards a lower risk of revision for dislocation but no overall reduced revision risk compared with the PLA"
Dooren-2023	Cohort study	Netherlands	January 2014 to January 2021	22959	343	22616	Patients who received a primary non-metal-on-metal (MoM)THA for OA using the DSA, PLA, or DAA.	Total hip arthroplasty	12	"The study showed no clinically meaningful differences between the DSA and either PLA or DAA"
Duijnisveld 2020	Cohort study	Netherlands	January 2016 to May 2017	104	52	52	Patients with primary osteoarthritis or osteonecrosis and a Body Mass Index (BMI) under 35 undergoing THA using DSA or MPA.	Total hip arthroplasty	12	"The DSA can be safely introduced as no learning curve in the prosthesis position, or the complication rate was found"
Hong 2025	Cohort study	Republic of Korea	January 2016 to December 2022	278	139	139	Patients who underwent a primary THA for the diagnosis of osteonecrosis of the femoral head and primary or secondary osteoarthritis.	Total hip arthroplasty	12	"The DSA was associated with lower patient-reported pain and a marked reduction in opioid consumption, delirium, and length of hospital stay"
Hu 2023	Cohort study	China	February 2020 to March 2021	48	24	24	Patients aged over 75 years with femoral neck fractures, Garden type III or IV, who underwent first-time unilateral hemiarthroplasty.	Hemiarthroplasty	6	"DSA is less invasive and has better clinical outcomes, which can allow an early return to daily living activities in elderly patients with displaced femoral neck fractures undergoing hemiarthroplasty"
Kenanidis 2023	Cohort study	Greece	January 2018 to May 2019	200	100	100	Adult patients with primary osteoarthritis and an ASA score ⩽3 undergoing primary unilateral THAs. Severe hip pain and disability of walking were the primary indications for THA.	Total hip arthroplasty	12	"The DSA approach may provide earlier functional recovery and hospital discharge for THA patients compared with SPA. DSA was equivalent to SPA concerning pain and blood loss, showing minimal complication rates"
LeRoy 2020	Cohort study	USA	January 2015 to January 2017	676	403	273	Patients underwent a primary THA for the diagnosis of osteoarthritis, avascular necrosis, or posttraumatic arthritis.	Total hip arthroplasty	24	"The superior approach to THA was associated with a significantly shorter length of hospital stay and a lower rate of discharge to rehab than the posterior approach. This approach can be used as a safe, minimally invasive, and tissue-sparing variation of a standard posterior approach for THA and has promising early outcomes"
Nam 2017	Cohort study	USA	–	238	42	196	Patients between the ages of 18 and 70 years undergoing an elective, primary THA for a diagnosis of osteoarthritis.	Total hip arthroplasty	–	"The study was unable to demonstrate a difference in the incidence of residual pain after use of a DSA or an MPA approach after THA"
Siljander 2020	Cohort study	USA	July 2013 to November 2017	3495	333	3162	Patients underwent elective primary THA with a DA, PL, or DS hip approach.	Total hip arthroplasty	3	"The study helps confirm that the DA, PL, and the DS approaches can all be effectively used with no significant overall differences in complication rates in the early postoperative period"
Ulivi 2021	RCT	Italy	April 2017 to December 2018	50	25	25	Patients aged between 60 and 75 with noninflammatory degenerative joint disease, rheumatoid arthritis, BMI between 18 and 30, and absence of contralateral THA.	Total hip arthroplasty	6	"DSA showed longer surgical time and lower blood loss compared with PL and early improvements in TUG, spatiotemporal, and kinematic gait parameters, highlighting rapid muscle strength recovery"
Wei 2025	RCT	China	January 2021 to June 2021	65	32	33	Patients aged 20–80 years planned for unilateral primary THA, with primary or secondary hip osteoarthritis or femoral head necrosis, a body mass index (BMI) under 40 kg/m^2^, and no severe systemic diseases or surgical contraindications.	Total hip arthroplasty	DSA:39.8 PA:40.1	"DSA demonstrated benefits in terms of reduced blood loss, improved pain scores, shorter incisions, and earlier ambulation. These advantages support the use of DSA for promoting early recovery and better mid-term functional outcomes"
Kamo 2024	Cohort study	Japan	June 2013 to May 2018	68	34	34	Patients with primary femoral neck fracture with a healthy opposite hip joint	Hemiarthroplasty	DSA:36.74 PA:28.51	SA-HA can be performed without torsion of the muscles and ligaments around the hip joint. Early recovery of walking ability and BI was a significant feature of SA-HAs.

Abbreviations: DSA: The direct superior approach, PA: posterior approach.

**Table 2:** Baseline characteristics of the included studies.

Study ID	Groups	N	Age (Y), mean (SD)	Sex (male), N (%)	BMI (kg/m2), mean (SD)	Diabetes mellitus, N (%)	Smoking, N (%)	ASA score, N (%)	
								1–2	3
Dooren 2023	DSA	1341	–	496 (37)	–	–	153 (11)	1099 (82)	–
	PA	117576	–	41758 (36)	–	–	12748 (11)	93073 (79)	–
Dooren-2023	DSA	343	–	120 (35)	–	–	35 (10)	292 (85)	–
	PA	22616	–	8655 (38)	–	–	2027 (9)	18330 (81)	–
Duijnisveld 2020	DSA	52	69 (8.4)	24 (46)	25 (3.4)	–	5 (10)	48 (92)	4 (8)
	PA	52	69 (8.4	18 (35)	25 (2.7	–	8 (15)	48 (92)	4 (8)
Hong 2025	DSA	139	58 (13)	72 (52)	24.5 (3.9)	19 (13.7)	28 (20)	111 (80.1)	28 (19.8)
	PA	139	58 (14)	78 (56)	24.5 (3.3)	20 (14.4)	37 (26)	119 (85.5)	20 (14.5)
Hu 2023	DSA	24	84.54 (2.11)	13 (54.17)	20.73 (1.47)	–	–	–	–
	PA	24	84.92 (2.15)	14 (58.33)	20.14 (1.02)	–	–	–	–
Kenanidis 2023	DSA	100	65.39 (8.38)	42 (42)	28.38 (3.09)	36 (36)	–	94 (94)	6 (6)
	PA	100	65.51 (7.85)	37 (37)	27.94 (2.98)	26 (26)	–	93 (93)	7 (7)
LeRoy 2020	DSA	403	63.4 (9.2)	190 (47.1)	28.1 (5.1)	–	–	359 (89.1)	44 (10.9)
	PA	273	63.4 (10.4)	138 (50.5)	31.6 (6.6)	–	–	212 (77.7)	61 (22.3)
Nam 2017	DSA	42	63.9 (6.1)	–	–	–	–	–	–
	PA	196	49.9 (7.1)	–	–	–	–	–	–
Siljander 2020	DSA	333	62 (11)	154 (46.2)	28.8 (5.3)	47 (14.1)	52 (15.6)	–	–
	PA	3162	64 (11)	1362(43.1)	30.2 (6.2)	395 (12.5)	338 (10.7)	–	–
Ulivi 2021	DSA	25	74 (8.9)	7 (28)	23 (2.8)	–	–	–	–
	PA	25	72 (7.7)	10 (40)	24 (2)	–	–	–	–
Wei 2025	DSA	32	47.3 (14)	15 (46.88)	23.2 (3.3)	–	–	25 (83.3)	5 (16.7)
	PA	33	53.7 (18.8)	17 (51.5)	22.9 (3.5)	–	–	26 (86.7)	4 (13.3)
Kamo 2024	DSA	34	80.7 (9.5)	8 (17.6)	20.5 (3.8)	–	–	–	–
	PA	34	84.1 (5.8)	7 (20.6)	20.3 (3.2)	–	–	–	–

### 8.3 Quality assessment

**Fig. 2:**
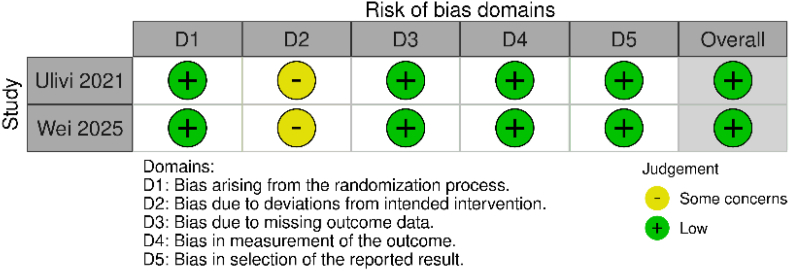
Risk of bias of the randomized controlled trials.

Quality assessment of the observational studies (n = 10) utilizing Newcastle-Ottawa Scale revealed predominantly robust methodological integrity, with nine studies demonstrating high-quality metrics and one exhibiting moderate quality (Table 3). Both randomized controlled trials underwent Cochrane Risk of Bias 2 evaluation, displaying minimal susceptibility to systematic error across assessed domains, as graphically represented in Fig. 2.

**Table 3:** Risk of bias assessment for the observational studies using the New-Castle-Ottawa Scale (NOS).

Study ID	Selection				Comparability	Outcome			
	Representativeness of the exposed cohort	Selection of the non-exposed cohort	Ascertainment of exposure	Demonstration that the outcome of interest was not present at the start of the study	Comparability of cohorts on the basis of the design or analysis	Assessment of outcome	Was the follow-up long enough for outcomes to occur	Adequacy of follow-up of cohorts	Overall quality
Dooren 2023	∗	∗	∗		∗∗	∗	∗	∗	Good
Dooren-2023	∗	∗	∗		∗∗	∗	∗	∗	Good
Duijnisveld 2020	∗	∗	∗		∗∗	∗	∗	∗	Good
Hong 2025	∗	∗	∗		∗∗	∗	∗	∗	Good
Hu 2023	∗	∗	∗		∗∗	∗	∗	∗	Good
Kenanidis 2023	∗	∗	∗		∗∗	∗	∗	∗	Good
LeRoy 2020	∗	∗	∗		∗	∗	∗	∗	Good
Nam 2017	∗		∗			∗	∗		Moderate
Siljander 2020	∗	∗	∗		∗	∗	∗	∗	Good
Kamo 2024	∗	∗	∗		∗	∗	∗	∗	Good

## 9 Primary outcome

### 9.1 Duration of hospital stay

**Figure 3:**
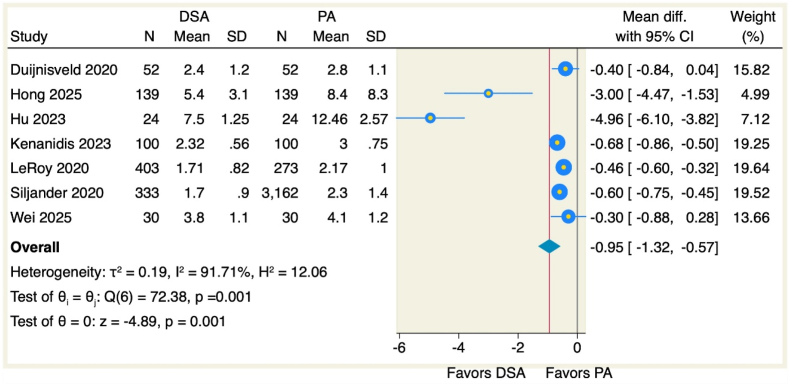
Forest plot of the duration of the hospital stay.

**Figure 4:**
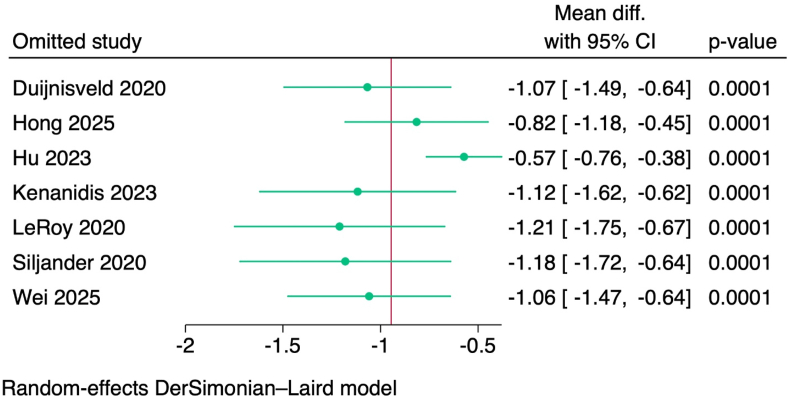
Leave-one-out test of the duration of the hospital stay.

**Figure 5:**
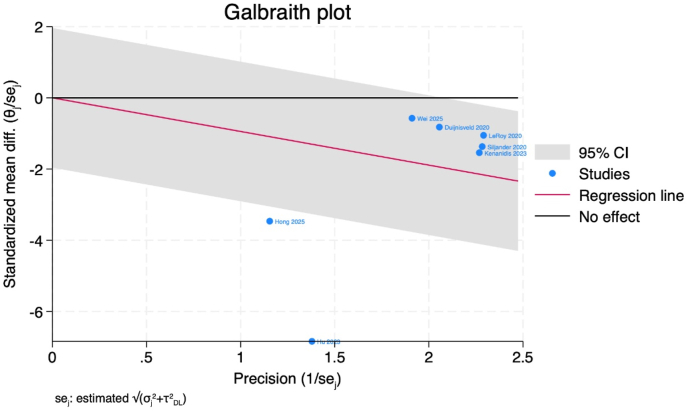
Galbraith plot of the duration of the hospital stay.

Length of hospitalization was evaluated across seven studies comprising 1081 patients who underwent direct superior approach and 3780 who received posterior approach. Meta-analysis demonstrated statistically significant reduction in hospitalization duration favoring DSA (MD -0.95 days, 95 % CI [−1.32, −0.57], p = 0.001) as illustrated in Fig. 3. Considerable statistical heterogeneity was observed (I^2^ = 91.71 %, p = 0.001). Sensitivity analysis through sequential omission of individual studies confirmed result stability, with effect estimates ranging from −0.57 (following Hu et al. 2 exclusion) to −1.21 (following LeRoy et al. 24 exclusion) as depicted in Fig. 4. Galbraith plot analysis identified two outlier investigations [2,12] contributing disproportionately to heterogeneity Fig. 5. Publication bias assessment via Doi plot revealed substantial asymmetry (LFK index −3.10), suggesting potential reporting bias (Supplementary Fig. 1).

## 10 Secondary outcomes

**Fig. 6:**
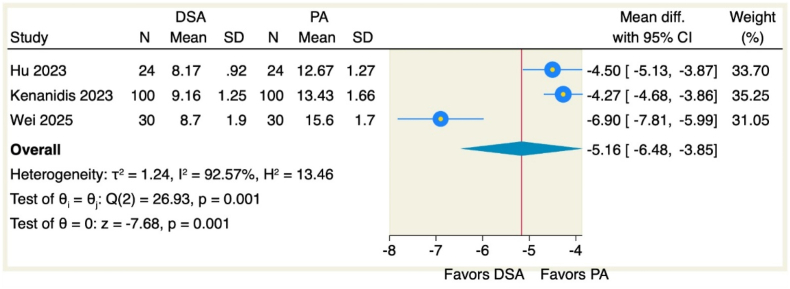
Forest plot of the incision length.

Our analysis of three pooled studies revealed significantly shorter incision lengths with the DSA approach compared to PA (MD = −5.16, 95 % CI [−6.48 to −3.85], p = 0.001), though with substantial heterogeneity (I^2^ = 92.57 %, p = 0.001), as illustrated in Fig. 6.

**Figure 7:**
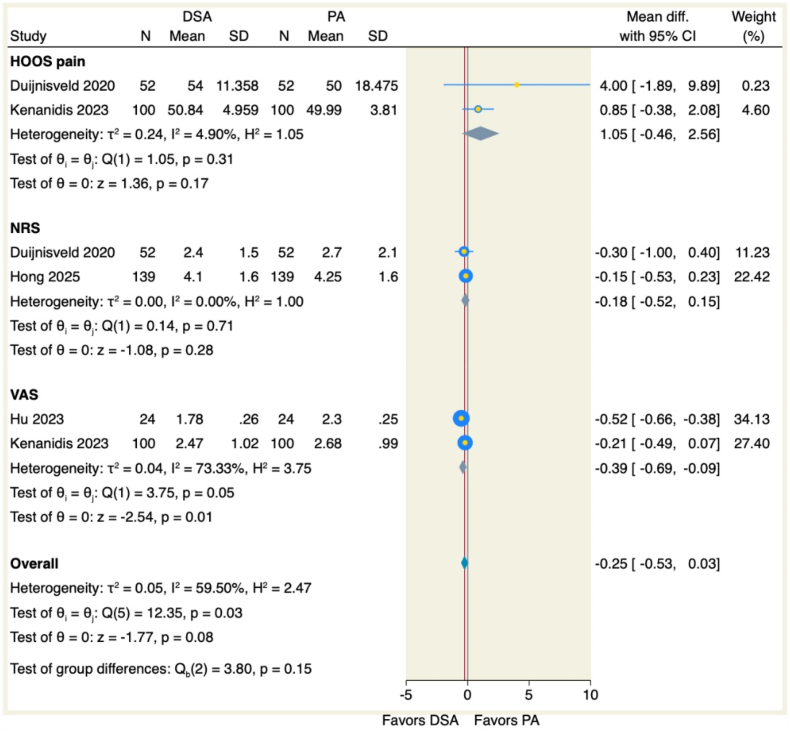
Forest plot of the pain scores.

**Fig. 8:**
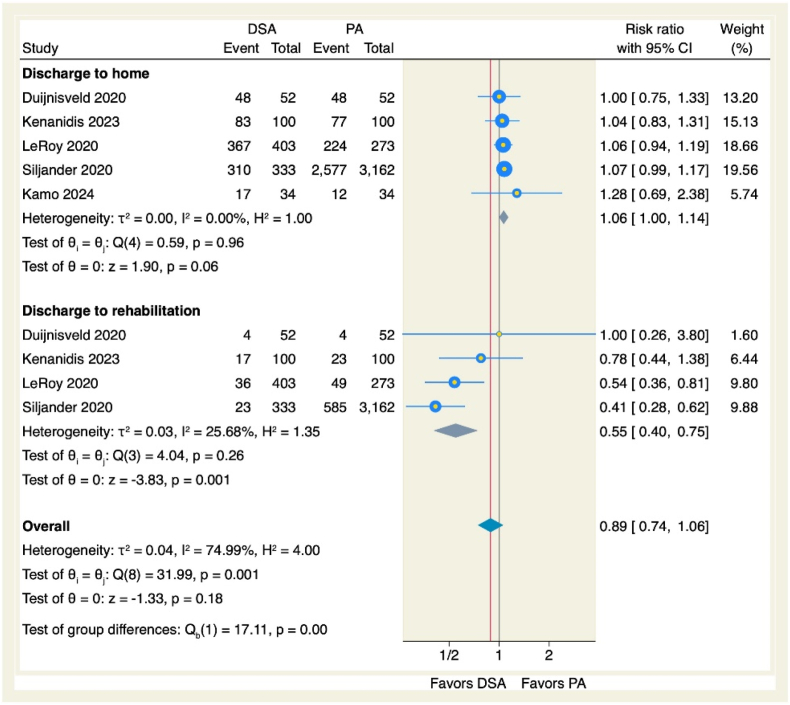
Forest plot of discharge.

Pain assessment using multiple validated tools showed no significant differences between approaches in HOOS pain subscales (MD = 1.05, 95 % CI [−0.46 to 2.56], p = 0.17; I^2^ = 4.90 %, p = 0.31) or NRS scores (MD = −0.18, 95 % CI [−0.52 to 0.15], p = 0.28; I^2^ = 0 %, p = 0.71). However, VAS scores were markedly lower with DSA (MD = −0.39, 95 % CI [−0.69 to −0.09], p = 0.01; I^2^ = 73.33 %, p = 0.05), as visualized in Fig. 7. Patients undergoing DSA demonstrated significantly reduced likelihood of rehabilitation discharge (RR = 0.55, 95 % CI [0.40 to 0.75], p = 0.001; I^2^ = 25.68 %, p = 0.26), yet home discharge rates remained comparable (RR = 1.06, 95 % CI [1 to 1.14], p = 0.07; I^2^ = 0 %, p = 0.96), as displayed in Fig. 8.

Our examination of surgical parameters across seven studies (n = 4696) found no significant differences in operative time between DSA and PA approaches (MD = 6.1, 95 % CI [−3.07 to 15.28], p = 0.19; I^2^ = 97.47 %, p = 0.001), regardless of procedure type - hemiarthroplasty (MD = 6.24, 95 % CI [−35.04 to 47.51], p = 0.77) or total hip arthroplasty (MD = 5.52, 95 % CI [−2.28 to 13.32], p = 0.17), as illustrated in Supplementary Fig. 2. Similarly, hematological outcomes remained equivalent, with no significant variations in hemoglobin levels (MD = 0, 95 % CI [−0.26 to 0.26], p = 1; I^2^ = 0 %, p = 1), as shown in Supplementary Fig. 3, blood loss (MD = −23.69, 95 % CI [−98.65 to 51.26], p = 0.54; I^2^ = 96.23 %, p = 0.001), as shown in Supplementary Fig. 4, or transfusion requirements (RR = 0.67, 95 % CI [0.42 to 1.07], p = 0.09; I^2^ = 11.04 %, p = 0.34), as illustrated in Supplementary Fig. 5.

Functional assessment using multiple validated instruments demonstrated comparable outcomes between techniques: HOOS daily activities (MD = 0.69, 95 % CI [−0.49, to 1.87], p = 0.25; I^2^ = 0 %, p = 0.25), HOOS quality of life (MD = −1.34, 95 % CI [−4.57 to 1.89], p = 0.42; I^2^ = 20.74 %, p = 0.26), HOOS symptoms (MD = −1.03, 95 % CI [−7.31 to 5.24], p = 0.75; I^2^ = 77 %, p = 0.04), and HOOS sport (MD = 2.32, 95 % CI [−1.89 to 6.52], p = 0.28; I^2^ = 0 %, p = 0.74), as displayed in Supplementary Fig. 6, HHS (MD = −0.32, 95 % CI [−1.01 to 0.37], p = 0.37; I^2^ = 0 %, p = 0.96), as shown in Supplementary Fig. 7, mHHS (MD = −0.73, 95 % CI [−6.59 to 5.13] p = 0.81; I^2^ = 57.58 %, p = 0.12), OHS (MD = 0.98, 95 % CI [−1.67 to 3.64], p = 0.47; I^2^ = 0 %, p = 0.93), and WOMAC (MD = −1.15, 95 % CI [−4.18 to 1.88], p = 0.46; I^2^ = 82.12 %, p = 0.02), as shown in Supplementary Figs. 8 and 9, respectively.

Revision rates remained statistically equivalent for all causes (RR = 1.03, 95 % CI [0.30 to 3.53], p = 0.97; I^2^ = 67.52 %, p = 0.02), including revisions due to hematoma and loosening (RR = 0.88, 95 % CI [0.43 to 1.81], p = 0.72; I^2^ = 0 %, p = 0.42), infection (RR = 1.34, 95 % CI [0.05 to 36.13], p = 0.86; I^2^ = 80.61 %, p = 0.02), or periprosthetic fracture (RR = 1.70, 95 % CI [0.26 to 10.99], p = 0.57; I^2^ = 79.41 %, p = 0.03), as illustrated in Supplementary Fig. 10.

Complication profiles showed no significant differences in dislocation (RR = 0.58, 95 % CI [0.25 to 1.33] p = 0.20; I^2^ = 0 %, p = 0.70), intraoperative fracture (RR = 1.16, 95 % CI [0.44 to 3.06] p = 0.77; I^2^ = 0 %, p = 0.89), periprosthetic fracture (RR = 1.54, 95 % CI [0.82 to 2.88], p = 0.18; I^2^ = 0 %, p = 0.94), death (RR = 0.79, 95 % CI [0.20 to 3.10], p = 0.73; I^2^ = 0 %, p = 0.97), infection (RR = 1.04, 95 % CI [0.27 to 4], p = 0.95; I^2^ = 0 %, p = 0.99), readmission (RR = 0.67, 95 % CI [0.24 to 1.82], p = 0.43; I^2^ = 45.96 %, p = 0.17), stroke (RR = 0.53, 95 % CI [0.05 to 6.13], p = 0.61; I^2^ = 0 %, p = 0.68), or thromboembolic events (RR = 1.20, 95 % CI [0.39 to 3.64], p = 0.75; I^2^ = 0 %, p = 0.93), as visualized **in** Supplementary Figs. 11, 12, and 13, respectively.

## 11 Discussion

Our meta-analysis, encompassing 147,098 patients, provides the most extensive and updated evidence comparing the DSA and PA approaches in patients undergoing HA. The DSA approach was notably associated with a lower hospital stay duration than the PA approach. Similarly, the DSA approach showed a shorter incision length, while no significant difference was detected between the two approaches regarding surgical time. Although pain outcomes measured by the HOOS and NRS scales revealed no significant difference, the DSA approach showed lower VAS scores relative to the PA. Furthermore, patients managed with the DSA technique were less likely to be discharged to rehabilitation. However, there was no difference between the two modalities regarding discharge to home. No substantial difference was detected in terms of blood-related outcomes, including hemoglobin level, blood loss, and the need for blood transfusion. In the same context, our pooled analysis did not favor either of the two approaches regarding functional outcomes measured by HOOS scores, HHS, OHS, and WOMAC. A similar rate of revision and postoperative complications involving dislocation, intraoperative fracture, infection, death, readmission, stroke, and thromboembolic events was noticed across both surgical modalities.

The DSA approach is considered a minimally invasive modification of the PA approach, aiming to achieve adequate surgical exposure and implant positioning with minimal tissue disruption.^4^ It involves an oblique angle incision ranging from 8 to 10 cm that begins from the posterior-proximal corner of the greater trochanter and extends proximally. An essential feature of the DSA technique is the muscle-sparing approach, which spares the iliotibial band, obturator externus tendon, and quadratus femoris muscle by separating the gluteal maximus bluntly in line with its fibres, which reduces the soft tissue trauma and enhances the postoperative stability, reduce hospitalization, and facilitate early functional recovery.^4^ This approach opposes the traditional posterior approaches, like the posterolateral approach, which usually includes releasing more external rotator muscles. The rationale for the muscle-sparing nature of the DSA modality is mainly driven by the understanding of the biomechanics of the hip joint, whereas the iliotibial band and certain short external rotators play a significant role in maintaining stability and facilitating early mobilization.^4^^,^^11^

Shin et al.^1^ conducted a meta-analysis comparing the DSA and PA approaches and finally included ten studies encompassing 28,063 patients who underwent THA. They found a significant advantage for DSA over PA regarding the perioperative outcomes encompassing hospital stay (SMD = −0.59, p < 0.01) and incision length (SMD = −2.75, p < 0.01), which aligned with our pooled analysis. Moreover, they found a greater benefit for DSA over PA in reducing blood loss (SMD = −0.26, p < 0.01) and the need for blood transfusion (OR = 2.32, p < 0.01). However, it is worth pointing out that Shin et al. employed the standardized mean difference (SMD), which allows them to pool the outcomes with different assessment tools and points. Although SMD enhances the statistical comparability, it has significant limitations when interpreting these results in the clinical context. For example, while they found a substantial benefit for DSA over PA regarding the hospital stay with a pooled SMD of −0.59, the lack of raw MD makes the clinical interpretation of these results difficult, whether it presents a reduction of several hours or multiple days. This limitation was addressed in our study as we pooled the continuous outcomes using MD instead of SMD, which provides a better clinical interpretability. Therefore, we recommend that the implementation of a more comprehensive approach in future studies by reporting both raw MD and SMD, to aid both the statistical robustness and clinical significance.

Notably, the minimally invasive approach of DSA was linked to major drawbacks, including prolonged surgical durations, restricted operative visibility, which may result in technical errors like component malposition, and an elevated risk of intraoperative complications such as periprosthetic fractures.^1^^,^^26^^,^^27^ This was not evident in our study, as we found no significant difference between DSA and PA regarding surgical time (MD = 6.1, p = 0.19). Notably, the comparable surgical time and the shorter learning curve attributable to the fact that DSA is a modification of PA, typically mastered in fewer than 20 cases, give an advantage for DSA over other technically challenging approaches, such as DAA.^9^^,^^10^ Additionally, previous studies showed reduced muscle and tendon damage with DSA compared to PA and DAA.^28^^,^^29^ This could justify our findings of a notably shorter incision length and lower VAS pain score among patients managed by the DSA compared to those who received PA.

Significantly, this tissue-sparing approach, DSA, did not heighten the risk of complications compared to PA. Our pooled analysis found no significant difference between the two approaches regarding complication rates, encompassing dislocation, intraoperative or periprosthetic fracture, infection, thromboembolic events, or readmission. Furthermore, no significant variations were detected in terms of all-cause surgery revision and revision across causes such as infection, periprosthetic fracture, or hematoma. In contrast, a recent population-based cohort study by Van Dooren et al.^18^ found a lower risk of surgery revision due to dislocation with DSA than PA (HR = 0.3, 95 % CI [0.1 to 0.9]) despite a higher number of small femoral heads in the DSA group than the PA group (29 % vs 20 %). This could be attributed to the better soft tissue preservation, including the capsule and quadratus femoris, and adequate implant positioning with DSA that probably mitigates the inherent instability risk associated with smaller femoral heads.^18^

Furthermore, the role of DSA in producing functional benefits is not well established yet. In a recent randomized trial by ULIVI et al.,^21^ the DSA group showed early functional benefits, as evidenced by higher Timed Up and Go (TUG) scores at three months postoperatively compared to the PL group. In the same context, the DSA group exhibited a lower hip rotation range of motion at one and three months than the PL group. Although this lower hip rotation range of motion might be a functional limitation, it may reflect better joint stability, promoting a safer early functional recovery. Our pooled analysis further supported this, which showed a lower discharge to rehabilitation for the 10.13039/501100021805DSA group than the 10.13039/100006131PA group. The improved joint stability potentially allowed for higher confidence in faster mobilization and safe discharge to home, thus mitigating the need for supervised rehabilitation services. These functional benefits of DSA could be attributed to its anatomical preservation of key stabilizing structures, including the fascia lata. The fascial system plays a substantial role in maintaining hip joint stability and sensorimotor integration. Disruption of fascia has been evidenced to compromise locomotor function owing to its integral role in connecting musculature and supporting proprioception.^30^^,^^31^ These findings highlighted the favorable effect of surgical approaches that preserve the fascia lata, such as the DSA, in providing better maintenance of the physiological coordination of the lower limb, thus enhancing stability and early recovery in the postoperative period.

## 12 Strengths and limitations

This systematic review and meta-analysis represent the most comprehensive and updated evidence evaluating the DSA compared to PA in HA. 10.13039/100014337Furthermore, including a large sample across different study designs and geographical regions supports our findings' external validity and real-world applicability. Moreover, using the raw mean difference to analyse outcomes further enhances the clinical interpretability of our results. Nevertheless, this study has various limitations. First, including observational studies could introduce selection bias and uncontrolled confounding that may weaken the robustness of our findings and statistical power. Second, considerable heterogeneity was noticed in several outcomes, which may be attributed to different surgical protocols and surgeon experience. Lastly, the follow-up duration in most studies was restricted to the early postoperative period, which hinders our ability to evaluate the long-term outcomes.

## 13 Implications in clinical practice and future suggestions

This meta-analysis offers notable clinical implications that could improve the decision-making process in hip arthroplasty. The DSA, as a muscle and fascial sparing modification of PA, exhibited significant perioperative benefits encompassing reduced hospitalization, shorter incision length, and lower VAS pain scores compared to PA. These advantages may contribute to early functional recovery and potentially lower healthcare costs. Furthermore, we found comparable complication rates and surgical times between DSA and PA, indicating that DSA could represent a safe substitute for PA without compromising surgical efficiency. Given that DSA is a modification of PA, with a familiar anatomical field, requiring a minimal learning curve, its implementation in clinical practice can be feasible, particularly in centres prioritizing faster postoperative recovery. Future researchers should focus on performing high-quality, large-sample size RCTs with extended follow-up durations to provide more robust evidence concerning the long-term outcomes of DSA compared to PA. Further examination of objective and subjective functional parameters is needed to better understand the functional impact of DSA.

## 14 Conclusion

This meta-analysis highlights the favorable role of DSA as an effective and safe alternative to PA in HA. Compared to PA, DSA demonstrated better perioperative outcomes, including reduced hospital stay, shorter incision length, and lower VAS pain scores. Without a significant increase in surgical time, DSA showed a lower need for postoperative rehabilitation than PA. However, we found no significant functional benefit for DSA over PA regarding functional scores, including HOOS, HHS, OHS, and WOMAC scales. Future high-quality research with longer follow-up durations focusing on these functional parameters is recommended to provide a clearer understanding of the role of DSA in the long-term functional outcomes.

## Guardian/patient's consent

N/A.

## Availability of data and materials

All data generated or analysed during this study are included in this published article [and its supplementary information files].

## Patient involvement

No.

## CRediT authorship contribution statement

Ralph Abdallah Conceptualization, Methodology, Formal analysis, screening, data extraction, quality assessment, Data curation, Writing – original draft, and Visualization. Mauz Asghar was involved in methodology development, screening, data extraction, quality assessment, Validation, and critical Writing – review & editing. Sadek Jaber Investigation, resource provision, and Writing – review & editing. Anthony Chalfoun and Ali Ghosn Investigation, Formal analysis, and manuscript review. Charbel Chaiban Data curation, quality assessment, and manuscript editing. Hadi Soukarieh Resources, Supervision, and contributed to quality assessment and manuscript revision. Ahmad Chokr Investigation, Resources, screening, and manuscript review. Maher Ghandour Supervision, Validation, and critical revision of the manuscript. Ümit Mert Conceptualization, Supervision, Project administration, and was responsible for manuscript review.

## Ethical statement

N/A.

## Funding statement

The study is not funded by any grant.

## Conflict of interest

The authors declare that they have no known competing financial interests or personal relationships that could have appeared to influence the work reported in this paper.

## References

[1] Shin K.-H., Kim J.-U., Jang I.-T. (2024). Early postoperative outcomes of the direct superior approach versus the posterior approach in total hip arthroplasty: a systematic review and meta-analysis. J Clin Med.

[2] Hu W., Xu W.-B., Li H. (2023). Outcomes of direct superior approach and posterolateral approach for hemiarthroplasty in the treatment of elderly patients with displaced femoral neck fractures: a comparative study. Front Surg.

[3] Hip Replacement Market Size (2025). https://www.fortunebusinessinsights.com/industry-reports/hip-replacement-implants-market-100247.

[4] Barrett A.A., Ezzibdeh R.M., Horst P.K., Roger D.J., Amanatullah D.F. (2019). Direct superior approach to the hip for total hip arthroplasty. JBJS Essential Surgical Techniques.

[5] Bergin P.F., Doppelt J.D., Kephart C.J. (2011). Comparison of minimally invasive direct anterior versus posterior total hip arthroplasty based on inflammation and muscle damage markers. J Bone Jt Surg Am Vol.

[6] Hoskins W., Bingham R., Lorimer M., Hatton A., de Steiger R.N. (2020). Early rate of revision of total hip arthroplasty related to surgical approach: an analysis of 122,345 primary total hip arthroplasties. J Bone Jt Surg Am Vol.

[7] Taunton M.J., Trousdale R.T., Sierra R.J., Kaufman K., Pagnano M.W. (2018). John charnley award: randomized clinical trial of direct anterior and miniposterior approach THA: which provides better functional recovery?. Clin Orthop Relat Res.

[8] Zijlstra W.P., De Hartog B., Van Steenbergen L.N., Scheurs B.W., Nelissen R.G.H.H. (2017). Effect of femoral head size and surgical approach on risk of revision for dislocation after total hip arthroplasty. Acta Orthop.

[9] Duijnisveld B.J., Van Den Hout J.A.A.M., Wagenmakers R., Koenraadt K.L.M., Bolder S.B.T. (2020). No learning curve of the direct superior approach in total hip arthroplasty. Orthop Surg.

[10] Ezzibdeh R.M., Barrett A.A., Arora P., Amanatullah D.F. (2020). Learning curve for the direct superior approach to total hip arthroplasty. Orthopedics.

[11] Wei Z., Xu Y., Zhu W., Weng X., Feng B. (2025). Direct superior approach versus posterolateral approach in mid-term clinical outcomes of total hip arthroplasty: a prospective randomized controlled study. BMC Muscoskelet Disord.

[12] Hong S.H., Yu K.H., Han S.B. (2025). Postoperative patient-reported pain and opioid consumption after total hip arthroplasty: a propensity score-matched comparison of the direct superior and posterior approaches. J Clin Med.

[13] Cochrane Handbook for Systematic Reviews of Interventions. (n.d.). Retrieved April 6, 2025, from https://training.cochrane.org/handbook.

[14] Page M.J., McKenzie J.E., Bossuyt P.M. (2021). The PRISMA 2020 statement: an updated guideline for reporting systematic reviews. BMJ (Clinical Research Ed.).

[15] Ouzzani M., Hammady H., Fedorowicz Z., Elmagarmid A. (2016). Rayyan—a web and mobile app for systematic reviews. Syst Rev.

[16] RoB 2: A revised Cochrane risk-of-bias tool for randomized trials | Cochrane Bias. (n.d.). Retrieved April 21, 2025, from https://methods.cochrane.org/bias/resources/rob-2-revised-cochrane-risk-bias-tool-randomized-trials.

[17] Ottawa Hospital Research Institute. (n.d.). Retrieved April 6, 2025, from https://www.ohri.ca/programs/clinical_epidemiology/oxford.asp.

[18] Van Dooren B., Peters R.M., Ettema H.B. (2023). Revision risk by using the direct superior approach (DSA) for total hip arthroplasty compared with postero-lateral approach: early nationwide results from the Dutch Arthroplasty Register (LROI). Acta Orthop.

[19] Van Dooren B., Peters R.M., Van Steenbergen L.N. (2023). No clinically relevant difference in patient-reported outcomes between the direct superior approach and the posterolateral or anterior approach for primary total hip arthroplasty: analysis of 37,976 primary hip arthroplasties in the Dutch Arthroplasty Registry. Acta Orthop.

[20] Siljander M.P., Whaley J.D., Koueiter D.M., Alsaleh M., Karadsheh M.S. (2020). Length of stay, discharge disposition, and 90-day complications and revisions following primary total hip arthroplasty: a comparison of the direct anterior, posterolateral, and direct superior approaches. J Arthroplast.

[21] Ulivi M., Orlandini L., Vitale J.A. (2021). Direct superior approach versus posterolateral approach in total hip arthroplasty: a randomized controlled trial on early outcomes on gait, risk of fall, clinical and self-reported measurements. Acta Orthop.

[22] Kenanidis E., Paparoidamis G., Pegios V.F., Anagnostis P., Potoupnis M., Tsiridis E. (2023). Earlier functional recovery and discharge from hospital for THA patients operated on via direct superior compared to standard posterior approach: a retrospective frequency-matched case-control study. HIP Int.

[23] Nam D., Meyer Z., Rames R.D., Nunley R.M., Barrack R.L., Roger D.J. (2017). Is the direct superior, iliotibial band-sparing approach associated with decreased pain after total hip arthroplasty?. J Arthroplast.

[24] LeRoy T.E., Hayden B.L., Desmarais J., Menendez M.E., Ward D. (2020). Early outcome comparison of the posterior approach and the superior approach for primary total hip arthroplasty. Arthroplasty Today.

[25] Kamo K. (2024). The superior approach in hemiarthroplasty for femoral neck fracture: a comparative analysis with the posterior approach. Hip & Pelvis.

[26] Berstock J.R., Blom A.W., Beswick A.D. (2014). A systematic review and meta-analysis of the standard versus mini-incision posterior approach to total hip arthroplasty. J Arthroplast.

[27] Lawson K.A., Ayala A.E., Larkin K., Seidel M.J. (2020). Anterior percutaneous-assisted total hip arthroplasty: surgical technique and early outcomes. Arthroplasty Today.

[28] Xiao C., Gao Z., Zhang S. (2021). Comparative prospective randomized study of minimally invasive transpiriformis approach versus conventional posterolateral approach in total hip arthroplasty as measured by biology markers. Int Orthop.

[29] Amanatullah D.F., Masini M.A., Roger D.J., Pagnano M.W. (2016). Greater inadvertent muscle damage in direct anterior approach when compared with the direct superior approach for total hip arthroplasty. The Bone & Joint Journal.

[30] Porr J. (2013). Fascia: the tensional network of the human Body. J Can Chiropr Assoc.

[31] (PDF) Analysis of the Christiania stop in professional roller hockey players with and without previous groin pain: A prospective case series study. (n.d.). ResearchGate. Retrieved April 21, 2025, from https://www.researchgate.net/publication/334113500_Analysis_of_the_Christiania_stop_in_professional_roller_hockey_players_with_and_without_previous_groin_pain_a_prospective_case_series_study.

