# Supplementary material for: Evaluating the direct superior approach compared to the traditional posterior approach for hip arthroplasty: A systematic review and meta-analysis
Source: J Orthop. 2025 May 27;68:219–29. doi: 10.1016/j.jor.2025.05.062 (PMC12169778; doi:10.1016/j.jor.2025.05.062)
Supplement: Multimedia component 1 [file mmc1.docx]

**
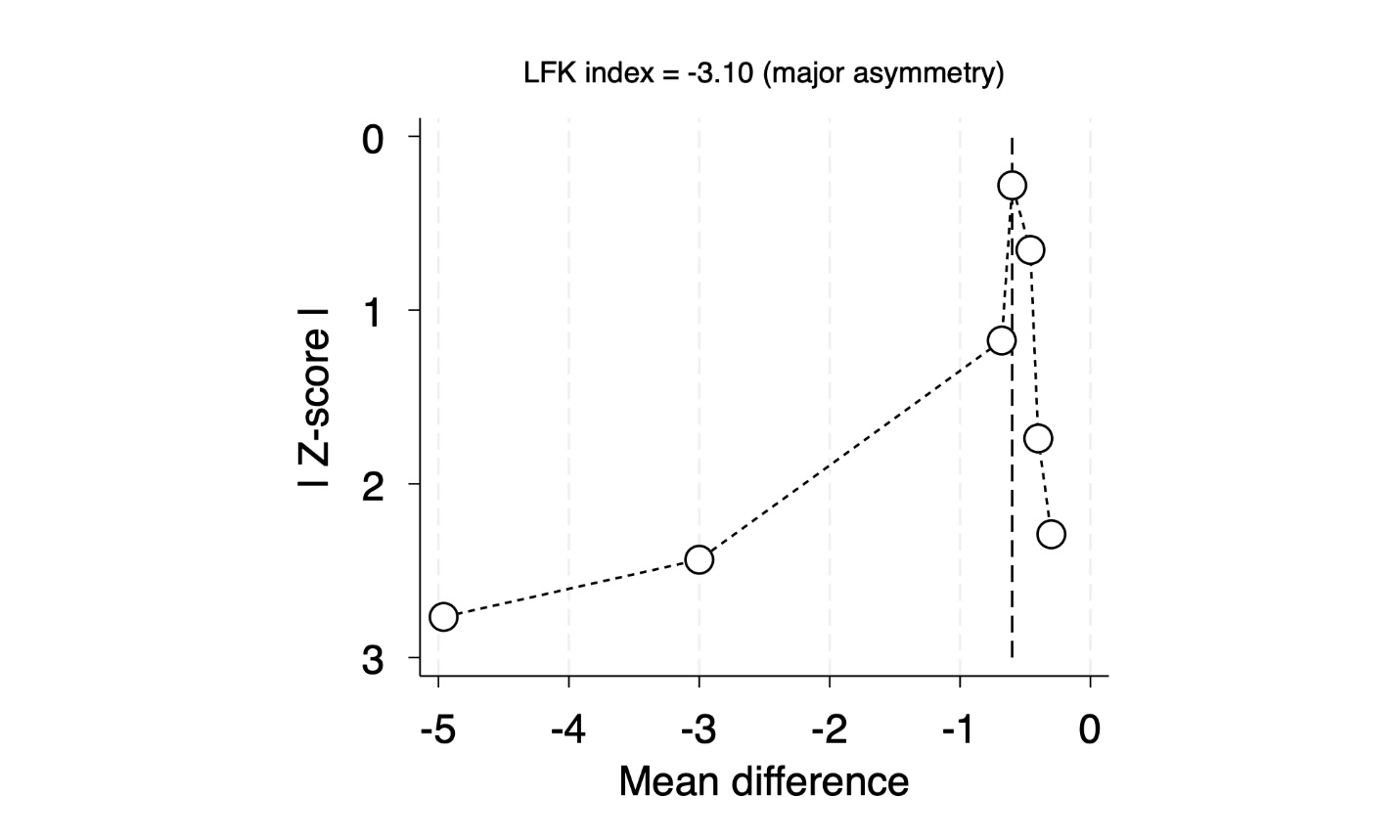

Supplementary Figure 1: Doi plot assessing publication bias in studies comparing hospital stay duration between direct superior approach (DSA) and posterior approach (PA)

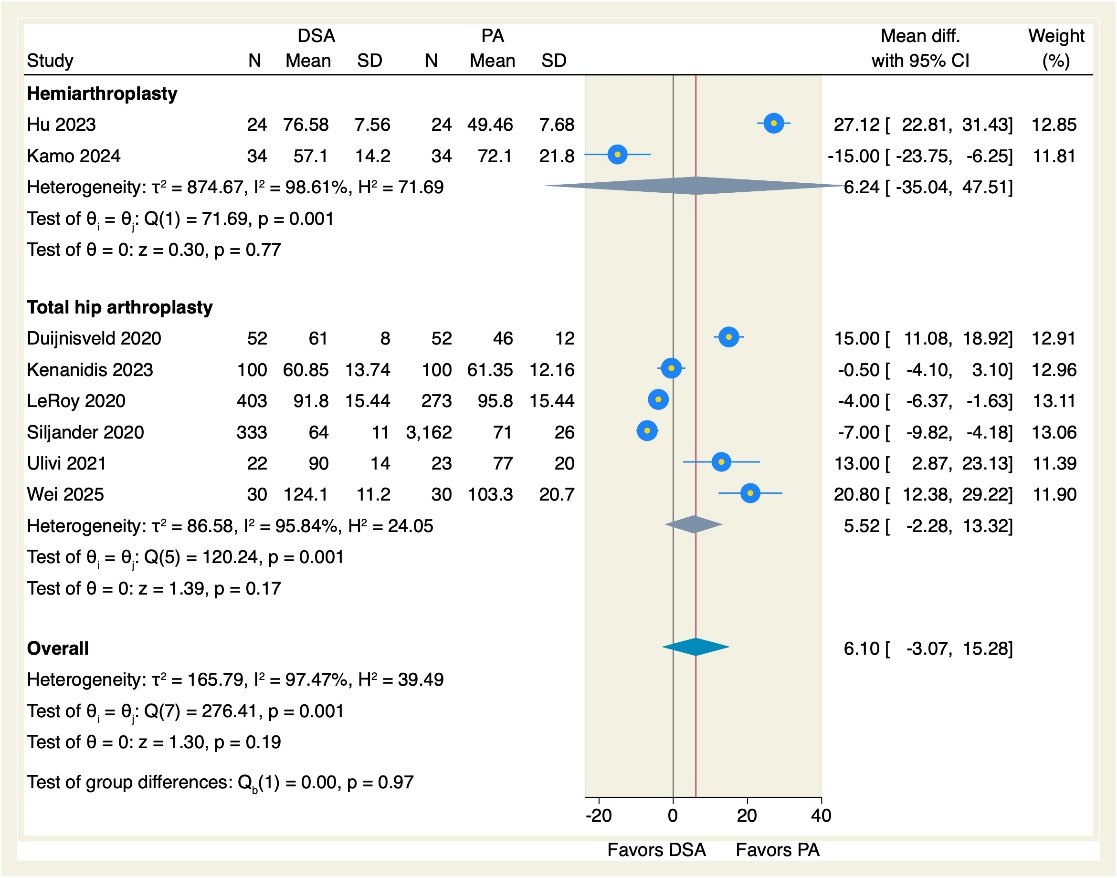

Supplementary Figure 2: Forest plot comparing operative time between direct superior approach (DSA) and posterior approach (PA)

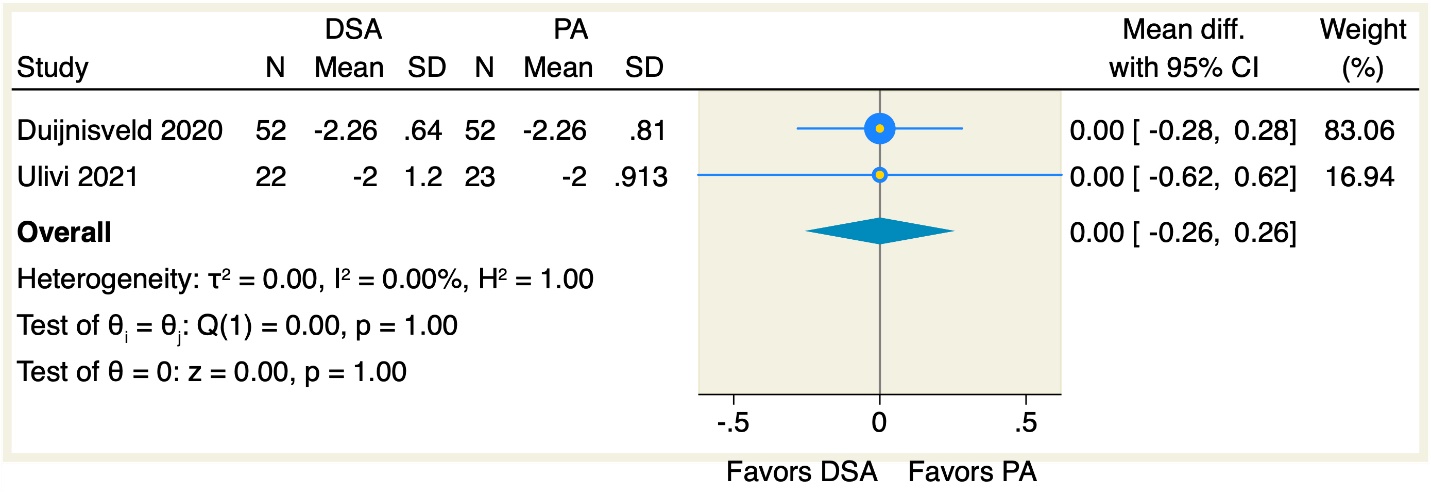

Supplementary Figure 3: Forest plot of hemoglobin level changes
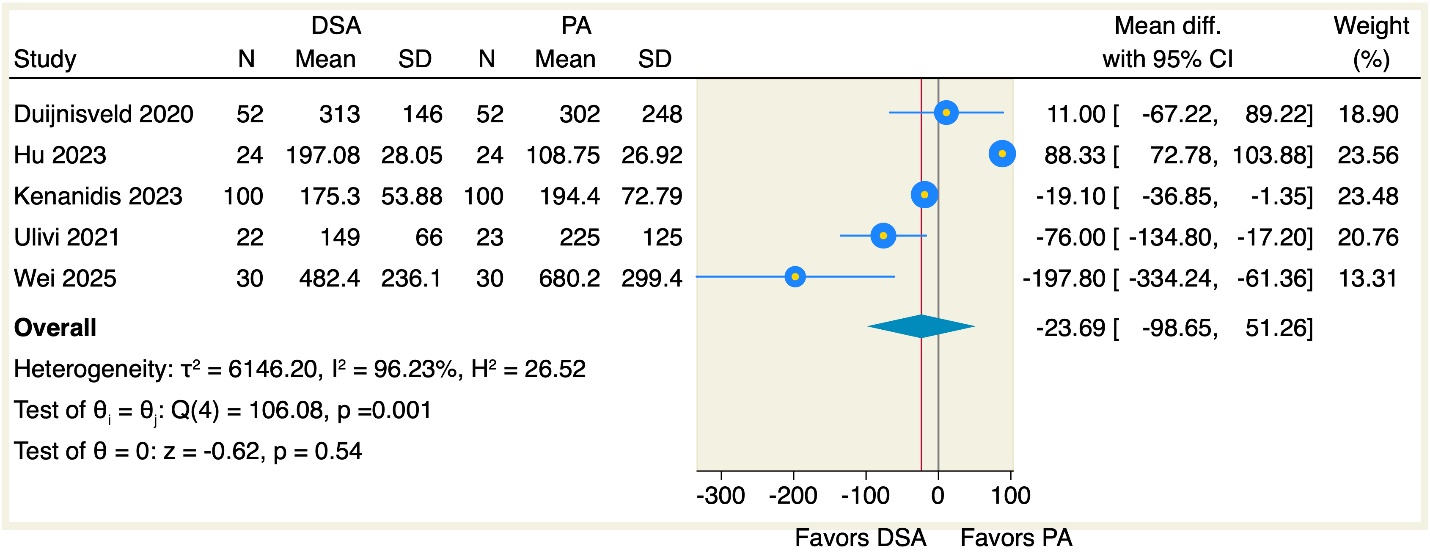

Supplementary Figure 4 : : Forest plot comparing estimated blood loss

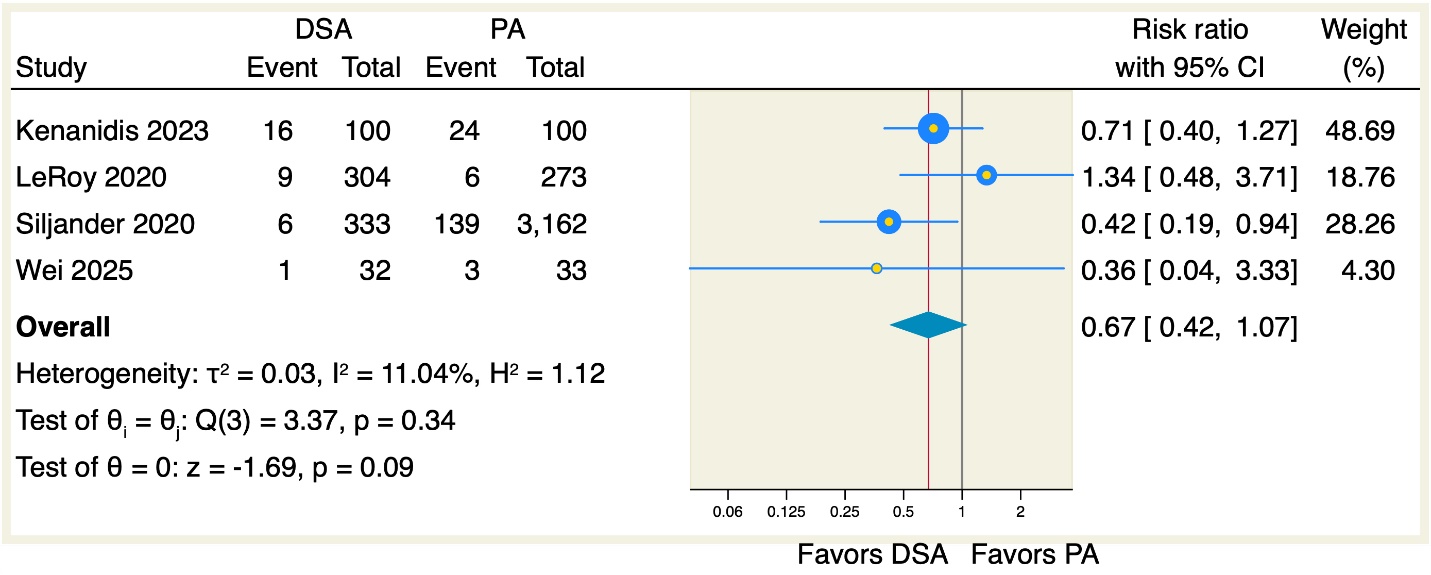

Supplementary Figure 5: Forest plot of transfusion requirements

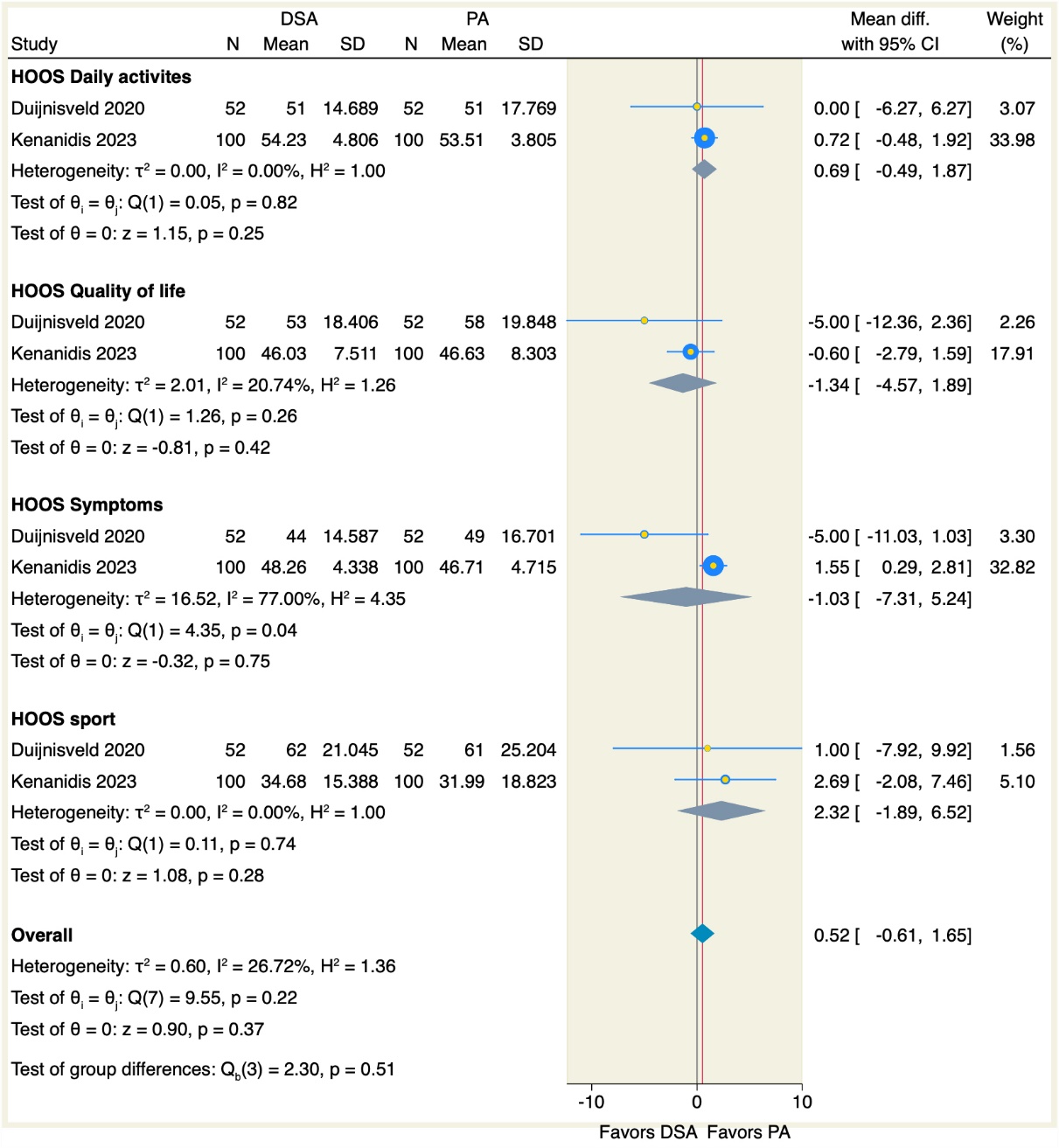

Supplementary Figure 6: Forest plots of HOOS subscale scores (daily activities, quality of life, symptoms, and sport)

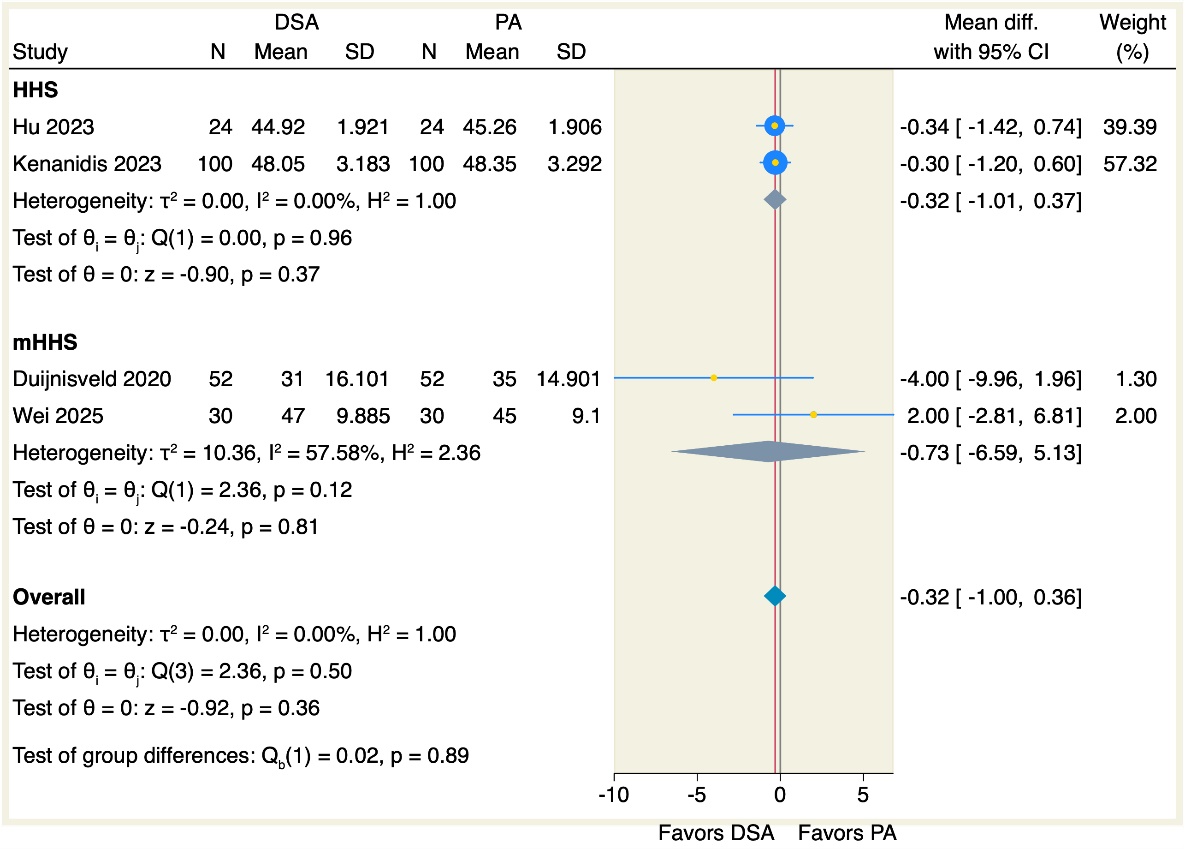

Supplementary Figure 7: Forest plot comparing Harris Hip Score (HHS) and modified Harris Hip Score (mHHS)

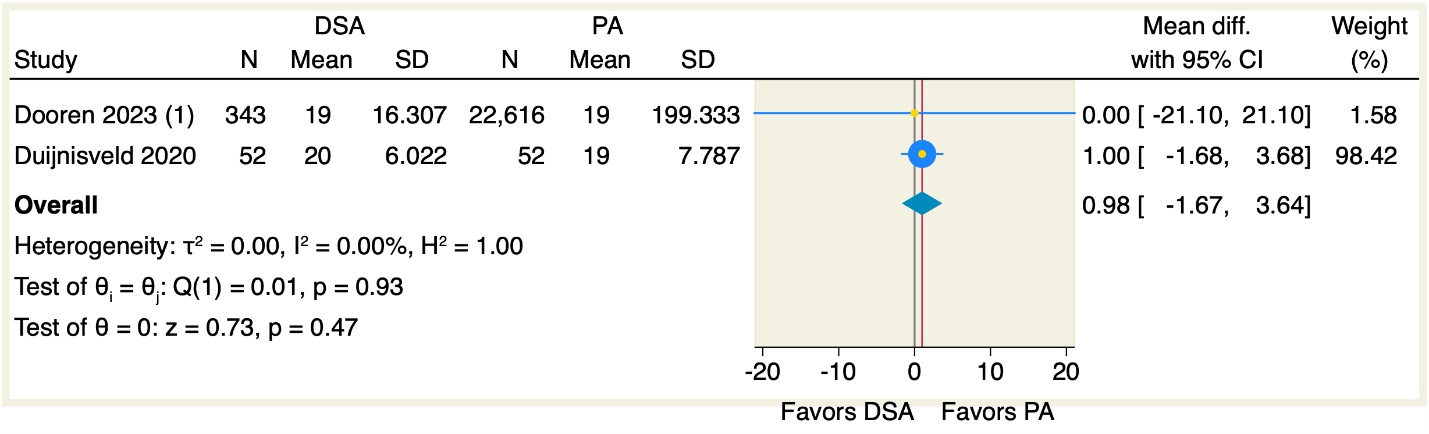

Supplementary Figure 8: Forest plot for Oxford Hip Score (OHS)

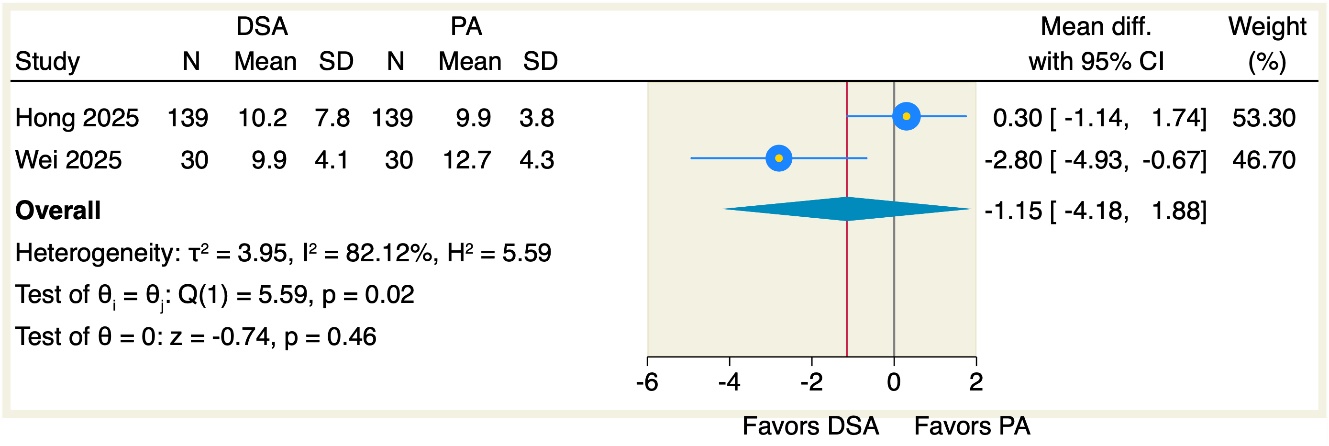

Supplementary Figure 9: Forest plot comparing WOMAC scores

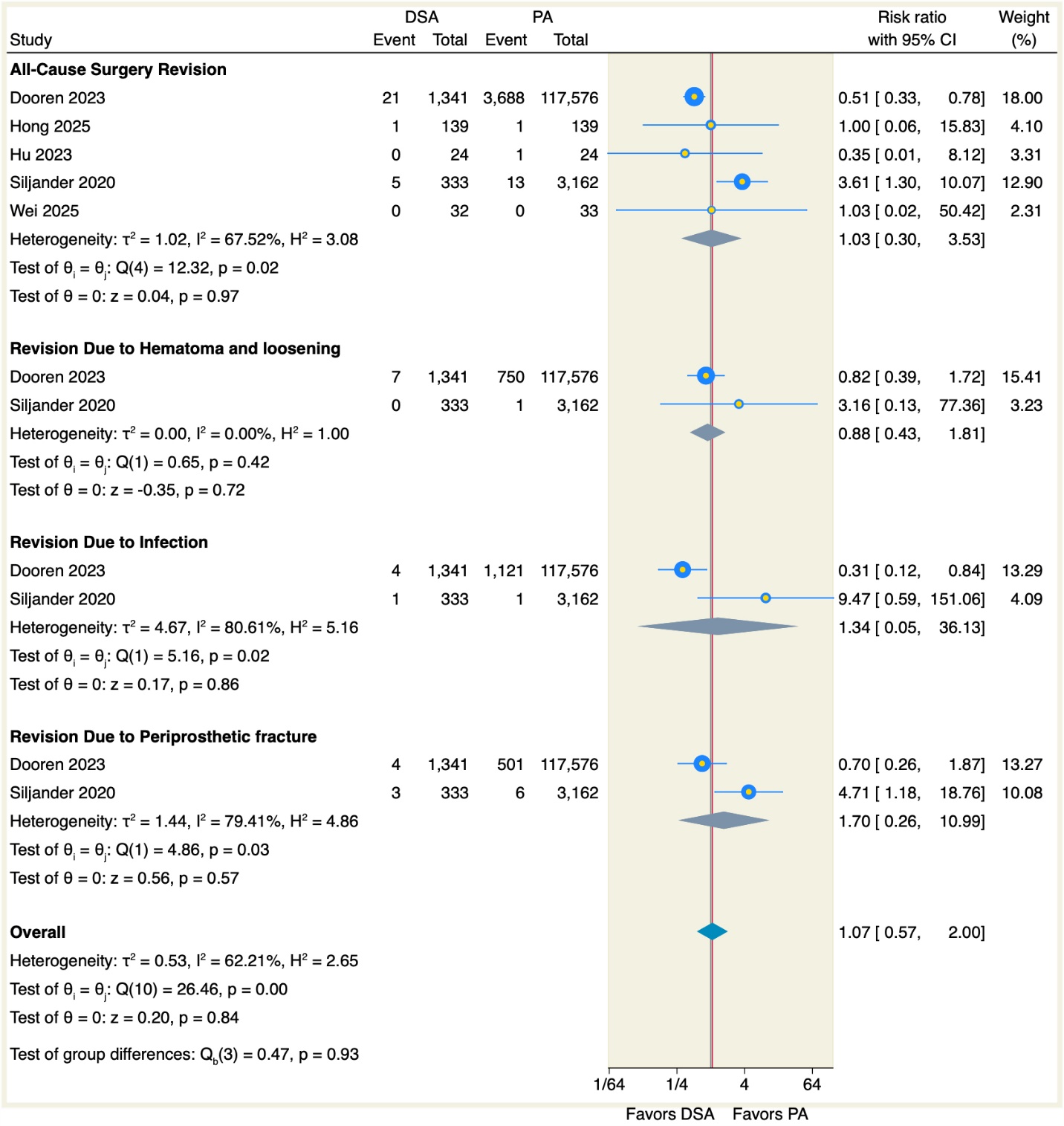

Supplementary Figure 10: : Forest plots of revision rates by cause (all-cause, hematoma/loosening, infection, and periprosthetic fracture)

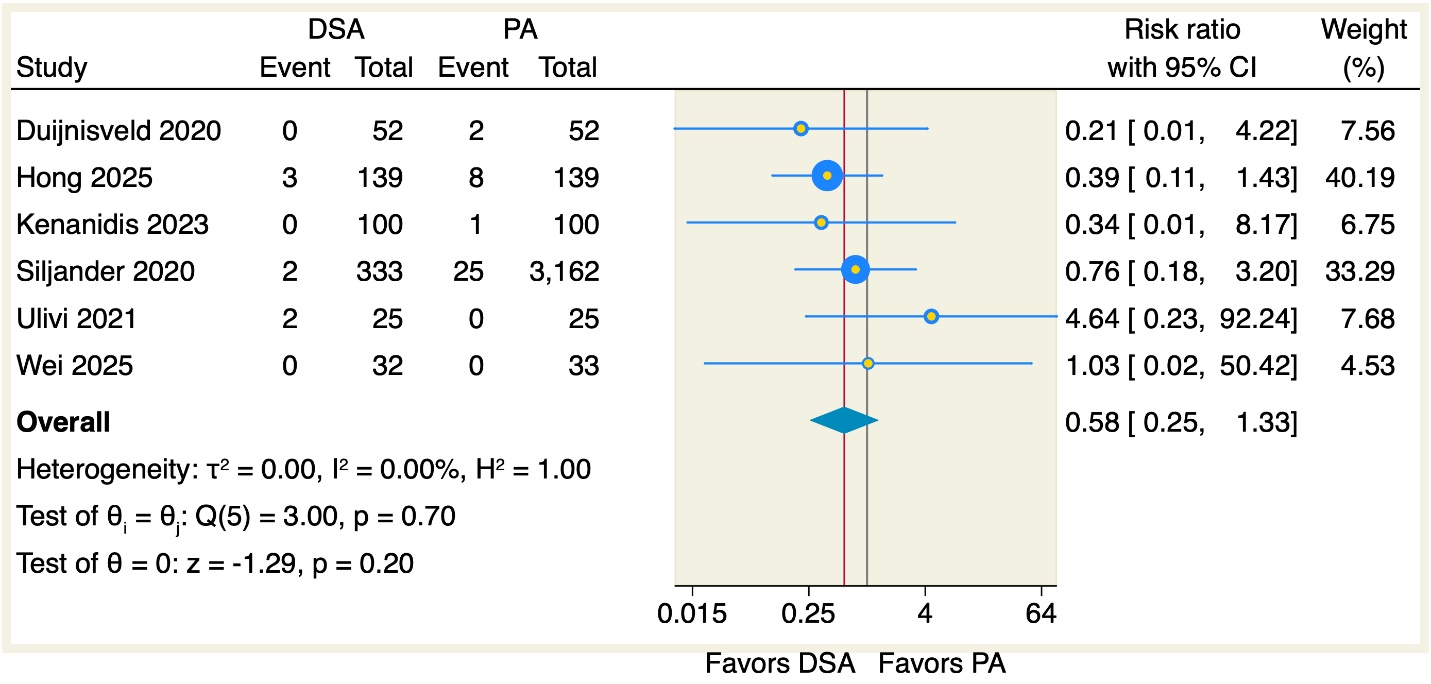

Supplementary Figure 11: Forest plot for dislocation rates

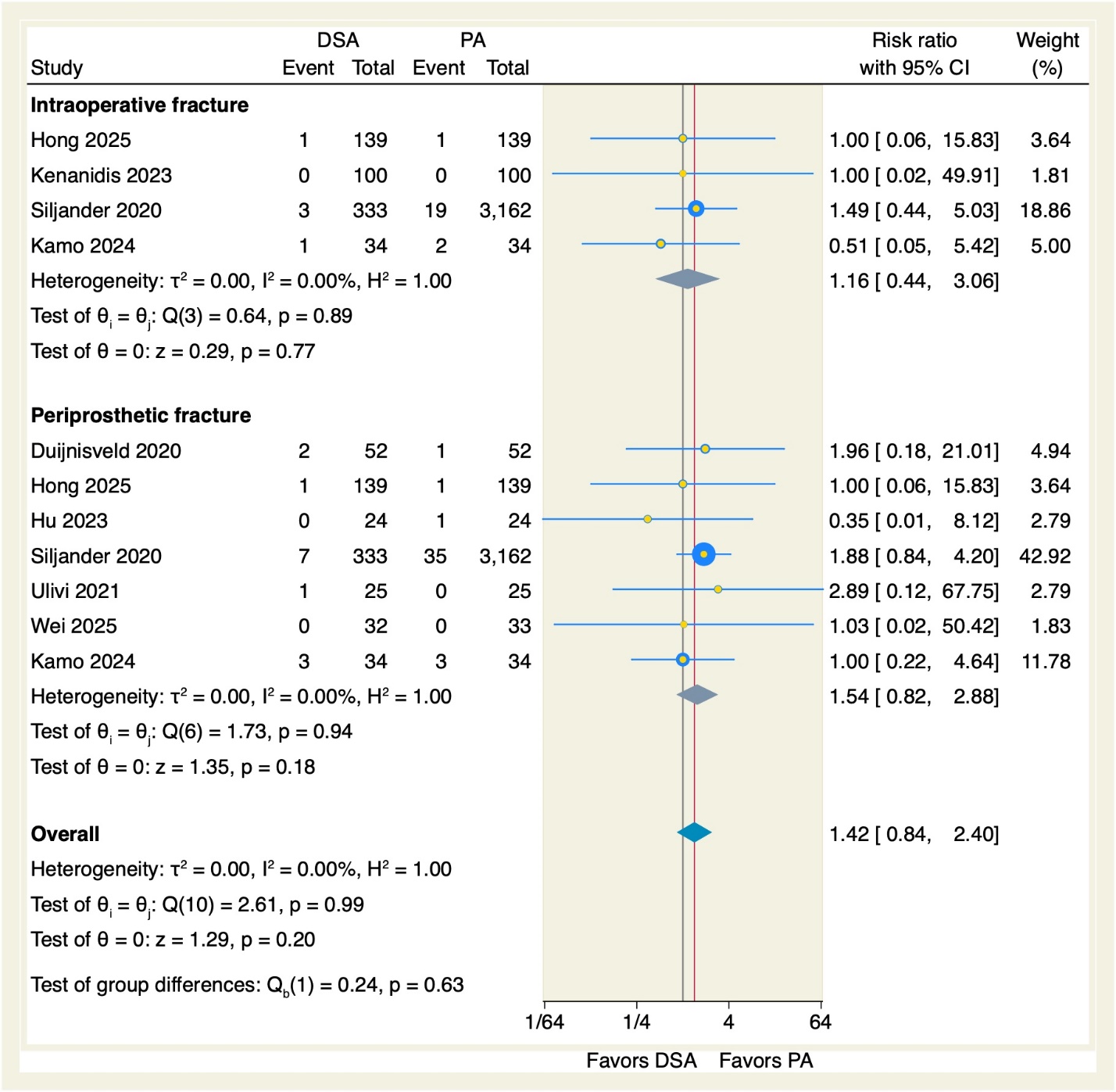

Supplementary Figure 12: Forest plots comparing rates of intraoperative fracture, and periprosthetic fracture

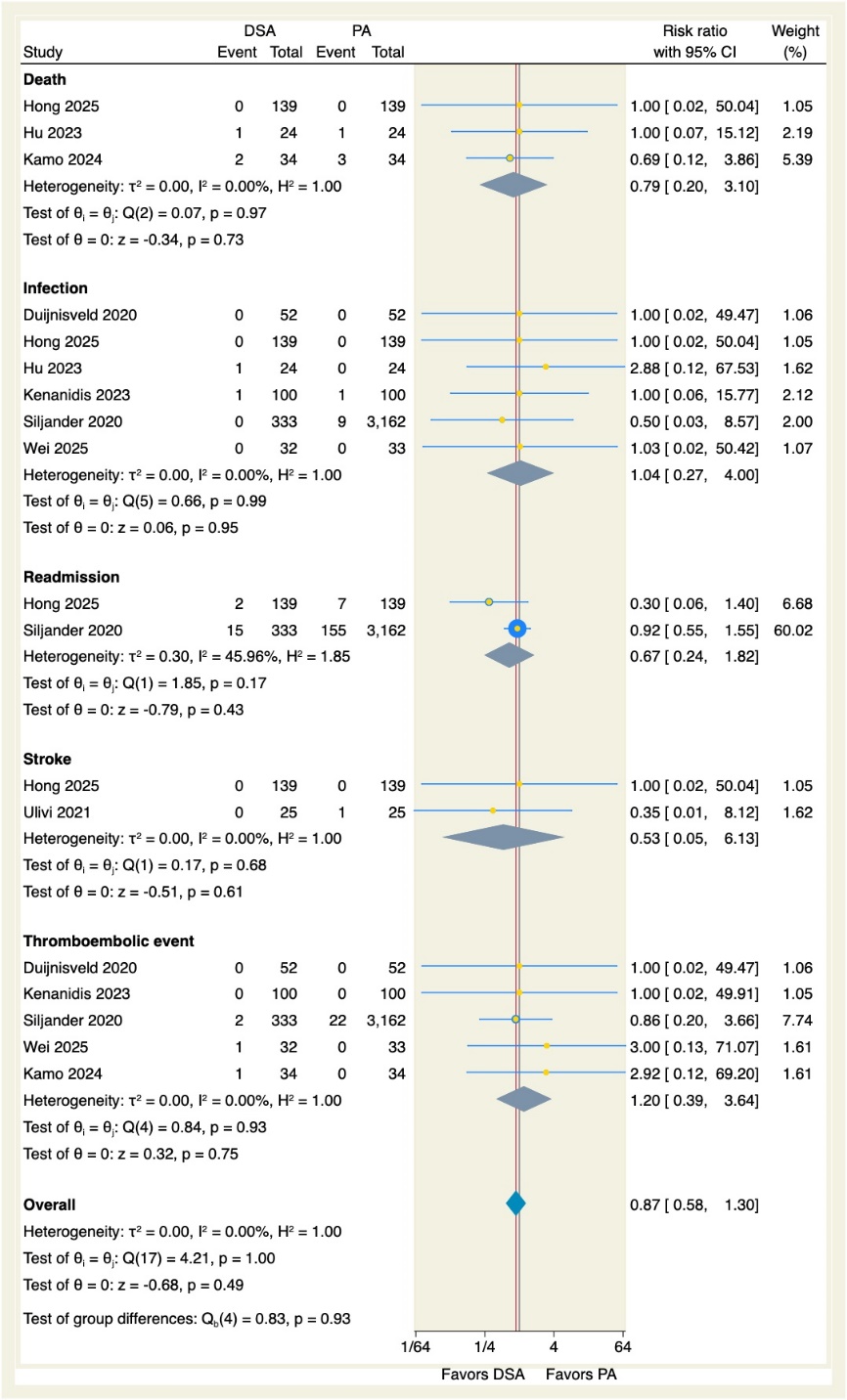

Supplementary Figure 13: Forest plots comparing mortality and infection, readmission, stroke, and thromboembolic events**

**Supplementary Figure 1: Doi plot assessing publication bias in studies comparing hospital stay duration between direct superior approach (DSA) and posterior approach (PA) for hip arthroplasty. Substantial asymmetry is observed (LFK index -3.10), suggesting potential reporting bias favoring DSA.**

**Supplementary Figure 2: Forest plot comparing operative time between direct superior approach (DSA) and posterior approach (PA) for hip arthroplasty. Subgroup analysis by procedure type (hemiarthroplasty vs. total hip arthroplasty) demonstrates no statistically significant differences between approaches (overall MD = 6.1 minutes, 95% CI [-3.07 to 15.28], p = 0.19) despite substantial heterogeneity (I² = 97.47%, p = 0.001).**

**Supplementary Figure 3: Forest plot of hemoglobin level changes comparing direct superior approach (DSA) and posterior approach (PA) for hip arthroplasty. No significant differences were detected between surgical approaches (MD = 0, 95% CI [-0.26 to 0.26], p = 1) with no observed heterogeneity (I² = 0%, p = 1).**

**Supplementary Figure 4: Forest plot comparing estimated blood loss between direct superior approach (DSA) and posterior approach (PA) for hip arthroplasty. No statistically significant differences were observed between approaches (MD = -23.69 mL, 95% CI [-98.65 to 51.26], p = 0.54) with substantial heterogeneity (I² = 96.23%, p = 0.001).**

**Supplementary Figure 5: Forest plot of transfusion requirements comparing direct superior approach (DSA) and posterior approach (PA) for hip arthroplasty. Analysis revealed comparable transfusion rates between surgical approaches (RR = 0.67, 95% CI [0.42 to 1.07], p = 0.09) with minimal heterogeneity (I² = 11.04%, p = 0.34).**

**Supplementary Figure 6: Forest plots of HOOS subscale scores (daily activities, quality of life, symptoms, and sport) comparing direct superior approach (DSA) and posterior approach (PA) for hip arthroplasty. No significant differences were detected across all functional domains, with heterogeneity ranging from none to moderate across subscales.**

**Supplementary Figure 7: Forest plot comparing Harris Hip Score (HHS) between direct superior approach (DSA) and posterior approach (PA) for hip arthroplasty. Analysis demonstrates comparable functional outcomes between approaches (MD = -0.32, 95% CI [-1.01 to 0.37], p = 0.37) with no heterogeneity (I² = 0%, p = 0.96).**

**Supplementary Figure 8: Forest plots comparing modified Harris Hip Score (mHHS) and Oxford Hip Score (OHS) between direct superior approach (DSA) and posterior approach (PA) for hip arthroplasty. Both functional assessment tools revealed statistically equivalent outcomes between surgical approaches.**

**Supplementary Figure 9: Forest plot comparing WOMAC scores between direct superior approach (DSA) and posterior approach (PA) for hip arthroplasty. Analysis shows comparable functional outcomes between approaches (MD = -1.15, 95% CI [-4.18 to 1.88], p = 0.46) with substantial heterogeneity (I² = 82.12%, p = 0.02).**

**Supplementary Figure 10: Forest plots of revision rates by cause (all-cause, hematoma/loosening, infection, and periprosthetic fracture) comparing direct superior approach (DSA) and posterior approach (PA) for hip arthroplasty. No statistically significant differences were observed across all revision categories.**

**Supplementary Figure 11: Forest plots comparing rates of dislocation, intraoperative fracture, and periprosthetic fracture between direct superior approach (DSA) and posterior approach (PA) for hip arthroplasty. All complications demonstrated statistically equivalent incidence between surgical approaches with minimal heterogeneity.**

**Supplementary Figure 12: Forest plots comparing mortality and infection rates between direct superior approach (DSA) and posterior approach (PA) for hip arthroplasty. Analysis revealed no significant differences in these major complications between surgical approaches (Death: RR = 0.79, 95% CI [0.20 to 3.10], p = 0.73; Infection: RR = 1.04, 95% CI [0.27 to 4], p = 0.95).**

**Supplementary Figure 13: Forest plots comparing rates of readmission, stroke, and thromboembolic events between direct superior approach (DSA) and posterior approach (PA) for hip arthroplasty. No statistically significant differences were observed in these systemic complications between surgical approaches.**
